# New Mechanisms of Action of Natural Antioxidants in Health and Disease

**DOI:** 10.3390/antiox9040344

**Published:** 2020-04-23

**Authors:** Silvana Hrelia, Cristina Angeloni

**Affiliations:** 1 Department for Life Quality Studies, Alma Mater Studiorum, University of Bologna, Corso d’Augusto 237, 47921 Rimini (RN), Italy; silvana.hrelia@unibo.it; 2 School of Pharmacy, University of Camerino, Via Gentile III da Varano, 62032 Camerino (MC), Italy

Natural antioxidants have been proposed to have beneficial effects on health and on different disease states, such as neurodegenerative and cardiovascular diseases, diabetes and cancer [1,2,3,4]. The use of natural plant antioxidant products to handle different diseases has very ancient roots; well before the development of modern medicine with synthetic drugs and antioxidants. A lot of the biological activities of natural antioxidants have been ascribed to their ability to scavenge reactive oxygen species (ROS) that counteract oxidative stress. In the last years, a multitude of studies have suggested that their classical hydrogen-donating antioxidant activity is unlikely to be the sole explanation for their effects [5]. First of all, natural antioxidants are subjected to an extensive metabolism in vivo that modifies their redox potentials. Moreover, the concentration of natural antioxidants and their metabolites in vivo are lower than that usually utilized in vitro. Accumulating evidence suggests that the cellular effects of natural antioxidants may also be mediated by their interactions with specific proteins central to intracellular signaling cascades [6,7,8], their modulation of the expression and activity of key proteins [9,10,11], their influencing of epigenetic mechanisms [12,13] or their modulation of the gut microbiota [14,15]. 

This special issue, concerning new mechanisms in the action of natural antioxidants in health and disease, contains nine contributions, seven research articles and two reviews, and details recent advances on this topic.

In recent years, increasing attention has been paid to natural dietetic antioxidants and their potential effect on human health. 

Corsetto et al. [16] focused on edible brown seaweeds, a rich source of natural antioxidants, extensively investigated for their ability to prevent and/or counteract different diseases [17,18]. In particular, the authors studied the possible mechanisms of *Fucus vesiculosus*’s antioxidant action and considered its bioactivity during the production of enriched rye snacks. They used a multiple-method approach, including chemical assays and cell-based bioassays, to characterize the potential mechanisms of the antioxidant action of seaweed extracts. They demonstrated that the antioxidant action of *Fucus vesiculosus* extracts is due to their high level of polyphenols, but also to their high Fe^2+^-chelating activity. Moreover, rye snacks enriched with *Fucus vesiculosus* showed a higher antioxidant potential, suggesting the use of these extracts to design functional foods.

Fermented foods are considered prominent constituents of the human diet because of their content in health-promoting compounds [19]. Fermentation is one the most ancient methods of food preparation, which increases the shelf life and improves the flavor of food matrices like soy, milk, meat, fruit and vegetables.

Fermented Papaya Preparation (FPP^®^) is a product obtained from the yeast fermentation of non-genetically modified Carica Papaya Linn, and has been shown to represent a valuable approach in obtaining systemic antioxidant effects. The study of Logozzi et al. [20] was aimed at verifying FPP^®^’s in vivo anti-aging effect, together with the modulation of the intracellular antioxidant system. Mice (C57BL/6J) were treated daily with FPP^®^ from either the 6th week or the 51st week of age. After a 10 month treatment, FPP^®^ led to an increase in telomeres length in the bone marrow and ovary, together with an increase in the plasmatic levels of telomerase activity and antioxidant levels, and a decrease of ROS. Interestingly, the treatment started at six weeks of age was more effective, suggesting a potential preventive role of FPP^®^ against molecular damages induced by age when started precociously.

Tonolo et al. [21] studied the antioxidant activity of different peptides extracted from fermented milk proteins. Bioactive peptides generated from milk can originate both from whey proteins and from caseins. Fermented milk was produced using Lactobacillus acidophilus NCFM®, Lactobacillus delbrueckii subs. bulgaricus and Streptococcus thermophilus. The authors identified four peptides, N-15-M, E-11-F, Q-14-R, and A-17-E, as the most effective against oxidative stress, and demonstrated that these bioactive peptides exert their antioxidant activity through the activation of the Keap1/ erythroid 2-related factor 2 (Nrf2) pathway.

Cardiovascular diseases are the leading cause of death in the world, and atherosclerosis, a chronic inflammatory process that involves a complex of pathophysiological effects, is one of the major risk factors. The development of atherosclerosis is related to the proliferation and migration of vascular smooth muscle cells (VSMCs) following stimulation with proinflammatory cytokines [22]. In recent years, phytochemicals have attracted considerable attention in the prevention and/or counteraction of atherosclerosis. The paper of Chou et al. [23] reported that a polyphenol-rich extract of *Hibiscus sabdariffa* leaves was able to inhibit matrix metalloproteinase expression and cell migration in VSMC A7r5 cells pretreated with TNF-a, by modulating protein kinase B (PKB) and inducing cell cycle G0/G1 arrest by inducing the expression of p53 and its downstream factors. The extract could also trigger a decrease in ROS production following TNF-a stimulation. In a well-established atherosclerotic New Zealand white rabbit model, the authors confirmed the in vitro data on the anti-atherosclerotic effect of *Hibiscus sabdariffa* leaf extract, suggesting that this extract could contribute to the protection against atherosclerosis and consequently against cardiovascular diseases.

Neurodegenerative disorders, like Alzheimer’s disease (AD), affect millions of people in the world and cause important memory impairment. Presently, there is no drug able to treat dementia-related afflictions, so phytochemical bioactive compounds are acquiring importance as a source of anti-AD agents. Capatina et al. [24], using a zebrafish (*Danio rerio*) model treated with 100 μM scopolamine (a muscarinic acetylcholine receptor blocker known to induce amnestic effects), investigated the potential antidepressant-like and cognitive-enhancing effect of *Rosmarinus officinalis* essential oil (REO). The authors demonstrated that REO prevents the anxiogenic-like effect of scopolamine, in a dose-dependent manner, and that it exhibited an anxiolytic and memory-enhancing profile. Moreover, REO treatment significantly reduced acetylcholinesterase activity, as compared to the scopolamine treated group, and counteracted the scopolamine-induced reduction in antioxidant enzyme activities in the zebrafish brain. Therefore, REO could be considered as a promising bioactive source for fighting cognitive performance-decrease and anxiety increase.

As previously underlined, the use of natural plant antioxidant products has very ancient origins, but nowadays research has made many steps forward, and the evolution of separative techniques has made it possible to identify specific active antioxidants compounds in natural sources, and develop them as potential therapeutic agents. 

In their review, Tan et al. [25] analyzed the function and mechanisms of cyanidin-3-glucoside and its phenolic metabolites in maintaining intestinal integrity. Cyanidin-3-glucoside is an anthocyanin, naturally present in black rice, black bean, purple potato and many berries. Cyanidin-3-glucoside is extensively metabolized in the gastrointestinal tract and gives rise to a series of secondary phenolic metabolites, including protocatechuic acid, phloroglucinaldehyde, vanillic acid and ferulic acid. Those metabolites have multiple effects, as they regulate the gut’s microbiota and modulate Nrf2 antioxidant and inflammatory pathways. Thanks to these effects, cyanidin-3-glucoside and its metabolites play a key role in maintaining intestinal integrity and function. 

In recent years, a lot of studies have underlined the synergic effect of different natural compounds when administered in combination [26]. On these bases, Marazzo et al. [27], using a three-dimensional neuronal cell culture, investigated the protective effect of three natural antioxidants (sulforaphane, epigallocatechin gallate, and plumbagin) alone or in combination, focusing on their activity against hydrogen peroxide-induced oxidative stress. Interestingly, the treatment with the combination of the three natural antioxidants was more effective than the treatments with the single compounds. In particular, the combined treatment positively modulated reduced glutathione (GSH), antioxidant enzymes (heme oxygenase 1, glutathione reductase and thioredoxin reductase) and insulin-degrading enzymes, and downregulated nicotinamide adenine dinucleotide phosphate (NADPH) oxidase 1 and 2, in respect to peroxide-treated cells. 

Franco et al. [28] reviewed the indirect antioxidant mechanisms of natural antioxidants, in particular hormesis, a mechanism that triggers the upregulation of enzymes essential for the innate detox pathways and/or the modulation of vitagenes expression [29]. Moreover, they suggested that natural antioxidants present in the chloroplast and mitochondria of plant cells may reach the mitochondria of mammalian cells, and make electron transport and oxidative phosphorylation more efficient. They concluded stressing the fact that, very often, in vitro antioxidant measures do not correlate with antioxidant action at the physiological level. 

Natural antioxidants have been widely studied also as effective topical natural cosmetic agents [30]. Hseu et al. [31] focused their attention on Ectoine, a natural extremolyte produced from several species of microorganisms under stressful conditions. Using UVA-irradiated human keratinocytes (HaCaT), they evaluated the effect of low Ectoine concentrations (0.5–1.5 μM) on depigmenting and anti-melanogenic parameters. They demonstrated that Ectoine treatment decreased ROS levels, α-melanocyte-stimulating hormone production, and proopiomelanocortin expression. Ectoine mediated the nuclear translocation of Nrf2 via p38, Akt and protein kinase C (PKC) pathways, and therefore the expression of antioxidant enzymes heme oxygenase-1, NAD(P)H dehydrogenase [quinone 1], and γ-glutamate-cysteine ligase catalytic subunit.

The conditioned medium, obtained from the Ectoine pre-treated and UVA-irradiated HaCaT cells, downregulated the tyrosinase, tyrosinase-related proteins and microphthalmia-associated transcription factor expressions in melanoma B16F10 cells, thus inhibiting melanin synthesis and evidencing a whitening effect. The authors concluded that Ectoine could be suggested as a potential and natural-based skin whitening agent to the cosmetic industry.

In conclusion, this special issue contributed to increasing the knowledge on the mechanisms of action of natural antioxidants, evidencing their pleiotropic role in the prevention and/or counteraction of degenerative diseases, and promoting also their application in the functional food and cosmetic field. 
